# *APOE *ε4 and Accelerated Cognitive Decline Among Cognitively Healthy Middle-Aged and Older Adults

**DOI:** 10.1001/jamanetworkopen.2026.0853

**Published:** 2026-03-06

**Authors:** Yu-Chu Ella Chung, Ren-Hua Chung, Chih-Cheng Hsu, Yu-Li Liu, Rai-Hua Lai, Wen-Jiu Hung, Ray-Chin Wu, Yun-Jin Jiang, Shao-Yuan Chuang, Shih Feng Tsai, Cheng-Chin Kuo, Chao Agnes Hsiung, Wei J. Chen

**Affiliations:** 1 Center for Neuropsychiatric Research, National Health Research Institutes, Miaoli, Taiwan; 2 Institute of Population Health Sciences, National Health Research Institutes, Miaoli, Taiwan; 3 National Center for Geriatrics and Welfare Research, National Health Research Institutes, Miaoli, Taiwan; 4 Institute of Molecular and Genomic Medicine, National Health Research Institutes, Miaoli, Taiwan; 5 Institute of Cellular and System Medicine, National Health Research Institutes, Miaoli, Taiwan

## Abstract

**Importance:**

Alzheimer disease (AD) pathology may begin decades before symptoms. Genetic factors, such as *APOE *ε4 carrier status and polygenic risk scores (PRS), influence AD risk, but their roles in cognitive decline among Asian populations remain unclear.

**Objective:**

To evaluate whether *APOE *ε4 carrier status and a non-*APOE* polygenic risk score (PRS_ADnapoe) are associated with age-related cognitive decline in community-dwelling older adults in Taiwan.

**Design, Setting, and Participants:**

This prospective cohort study used data from 2 assessment waves of the Healthy Aging Longitudinal Study in Taiwan, spanning 2009 to 2019. Participants were aged 55 years and older and had both genetic data and Mini-Mental State Examination (MMSE) scores. Data analyses were conducted from August to December 2025.

**Exposures:**

*APOE *ε4 carrier status (noncarrier, heterozygote, homozygote) and PRS_ADnapoe score, derived from genome-wide association summary statistics excluding *APOE* variants.

**Main Outcomes and Measures:**

The primary outcome was change in MMSE scores, which were assessed cross-sectionally and longitudinally, modeled with mixed-effects regression accounting for age-related effects and covariates including sex, education, smoking, and population structure.

**Results:**

Among 4392 participants (mean [SD] age, 68.2 [7.8] years; 2359 [53.7%] women), 723 (16.5%) were *APOE *ε4 heterozygotes and 33 (0.8%) were *APOE *ε4 homozygotes. Over a mean (SD) follow-up of 6.3 (0.9) years, the mean (SD) annual MMSE decline was −0.2 (0.5). *APOE ε4* carriage was associated with a significantly steeper quadratic age–associated decline in MMSE scores compared with noncarriers (estimate, −0.005; SE, 0.001; *P* = .001). This association was strongest among homozygotes (estimate, −0.017; SE, 0.008; *P* = .03), with MMSE trajectories diverging after approximately age 70 years. In contrast, PRS_ADnapoe scores were not associated with MMSE decline. Sensitivity analyses restricted to participants with 2-wave data and adjusted with inverse probability of censoring weighting confirmed these findings.

**Conclusions and Relevance:**

In this cohort study of middle-aged and older adults in Taiwan, *APOE *ε4 carriage, particularly homozygosity, was associated with accelerated age-related cognitive decline detectable after age 70 years, whereas non-APOE polygenic risk was not associated with cognitive decline over the current follow-up. These results highlight the potential utility of early genetic risk awareness and support consideration of targeted preventive strategies for *APOE *ε4 carriers.

## Key Points

**Question:**

Is *APOE* ε4 carrier status associated with accelerated cognitive decline in cognitively healthy adults in Taiwan, and does an *APOE*-excluded polygenic risk score estimate risk of similar decline?

**Findings:**

In this cohort study of 4392 adults aged 55 years and older, *APOE *ε4 carriers—especially homozygotes—showed accelerated decline in Mini-Mental State Examination scores, diverging from noncarriers after age 70 years. The polygenic risk score was not associated with cognitive decline during the 6-year mean follow-up period.

**Meaning:**

In this study, *APOE *ε4 carrier status was associated with early cognitive decline, highlighting midlife risk awareness and lifestyle interventions, while non-*APOE* polygenic risk may require longer follow-up to manifest.

## Teaser

This cohort study examines the association of *APOE *ε4 carrier status and cognitive decline among adults aged 55 years older in Taiwan and evaluates whether a polygenic risk score that does not include *APOE *status is associated with risk of cognitive decline.

## Introduction

Alzheimer disease (AD) is a leading cause of morbidity in later life and is characterized by progressive memory loss and loss of the ability to perform daily tasks. An estimated 57.4 million people lived with dementia worldwide in 2019, a number projected to triple by 2050, with AD as the most common etiology.^1^ Current pharmacologic therapies slow progression but do not reverse the disease. Amyloid-β (Aβ) pathology likely begins up to 2 decades before symptom onset.^2^ Consequently, AD is viewed as a continuum that spans a preclinical stage (asymptomatic with neuropathology), a prodromal stage (impairment in at least 1 cognitive domain with preserved function), and a dementia stage (multidomain impairment with functional decline).^3^ In this context, early cognitive change provides a pragmatic, measurable signal of preclinical disease.

AD is an age-related multifactorial neurodegenerative disorder^4^ with contributions from various risk factors, including metabolic factors and lifestyle choices,^5^ environmental exposures,^6^ and genetic predispositions.^2^ Genetic information can provide an early clue to future AD risk. Monogenic autosomal-dominant forms—due to pathogenic variants in Aβ protein precursor, presenilin-1, or presenilin-2— account for fewer than 1% of cases.^7^ In contrast, variants in apolipoprotein E (*APOE*) are common and contribute substantially to late-onset AD risk^7^; recent evidence further suggests that *APOE *ε4 homozygosity may represent a distinct genetic form of AD.^8^ *APOE *ε4 frequency varies across populations and is generally lower in Asia than in Europe and North America.^9^ In parallel, genome-wide association studies (GWAS) enable polygenic risk scores (PRS) that aggregate contributions from many common variants associated with AD risk.^10,11^ In settings with lower *APOE *ε4 prevalence, a PRS that excludes *APOE* variants (ie, PRS_ADnapoe) could augment risk stratification for AD.

A growing body of research has started to explore the relationship between genetic risk factors and changes in cognitive function in the preclinical stage.^12,13,14,15,16,17,18,19,20,21^ However, the evidence remains inconsistent, potentially due to factors such as limited education levels, wide age ranges, and variability in measurement tools.^3,22,23^ Importantly, very few studies have included Asian populations.^18^ This represents a significant gap, given potential ethnic differences in genetic architecture and environmental exposures that may influence cognitive aging.

To address these gaps, we investigated genetic risk and cognitive decline in a community-based cohort of adults aged 55 years and older from the Healthy Aging Longitudinal Study in Taiwan (HALST).^24^ This study aimed to (1) describe the distribution of *APOE *ε4 status and PRS_ADnapoe scores and the sociodemographic and comorbidity factors associated with them; (2) compare 2-wave cognitive change by *APOE *ε4 status and PRS_ADnapoe tertiles; and (3) examine whether *APOE *ε4 status and PRS_ADnapoe were associated with cognitive change over time using mixed-effects modeling.

## Methods

### HALST Cohort and Selection of Participants

HALST is a prospective community-based cohort initiated in 2009 to identify determinants of healthy aging in Taiwan.^24^ Participants aged 55 years or older were recruited from 7 nationally defined regions covering major urban and rural areas across northern, central, southern, and eastern Taiwan, using household registries and hospital catchment areas. This sampling framework was designed to capture participants with diverse sociodemographic backgrounds rather than to constitute a fully representative sample of the national elderly population.^24^ Participants were then followed up longitudinally. In wave 1 (2009-2013), 5664 participants were enrolled; 5349 (94.4%) completed home interviews and provided venous blood samples. Cognitive function was assessed using the Mini-Mental State Examination (MMSE) by trained interviewers during home interviews after excluding participants with self-reported clinical dementia diagnosis. Annual telephone follow-ups were conducted to briefly monitor participants’ health status. Approximately 74% (4170 participants) participated in wave 2 (2014-2019).

The summary of censoring and causes of attrition is shown in eFigure 1 in Supplement 1. In brief, 4392 participants were included for analysis. Given the relatively low average educational level and high proportion of illiteracy in the cohort, an MMSE cutoff of 16 has been used to distinguish clearly impaired individuals from those with low scores primarily due to limited schooling.^25^ The items that were not answered or could not be answered due to conditions including blindness, illiteracy, or upper-limb motor impairment were scored as 0. Participants with 6 or more missing items were considered incomplete.^26^ At wave 1, the number of missing items per person ranged from 0 to 5; 3235 (73.7%) had no missing items and none had more than 5 missing items. At wave 2, among 4392 participants, 2627 (59.8%) had no missing MMSE items, 632 (14.4%) had 1 to 5 missing items, 5 (0.1%) had 6 to 10 missing items, and 1128 (25.7%) had entirely missing MMSE data. Accordingly, 3259 participants were classified as having complete MMSE data across both waves. Of the 1133 participants with 6 or more missing items, 1128 (99.6%) had entirely missing MMSE data.

The HALST study protocol was approved by the Ethics Committee of the National Health Research Institute, Taiwan. All participants provided written informed consent. The current study followed the Strengthening the Reporting of Observational Studies in Epidemiology (STROBE) reporting guideline for cohort studies.

### Genotyping, Quality Control, and Imputation

Genome-wide genotyping used the TWB version 2.0 Affymetrix Axiom array, covering approximately 470 000 single nucleotide variants (SNVs). Quality control procedures excluded SNVs with call rates less than 95% or Hardy-Weinberg equilibrium of *P* < .0001.^27^ Samples with a call rate less than 95%, sex discordance, duplication (π̂ > 0.9), possible contamination, or close relatedness were excluded.^28,29,30^ All analyses were performed using PLINK version 1.9.^31^

Principal components (PCs) for population structure^32^ were derived using PC-AiR. Genotype imputation was performed via the Michigan Imputation Server with the 1000 Genomes reference panel.^33^ Variants with a minor allele frequency (MAF) greater than 1% and imputation quality metric (Rsq) greater than 0.3 were retained.

### PRS for Alzheimer Disease

PRS were computed using PRS-CS (a bayesian continuous-shrinkage method)^34^ with HapMap3 SNVs (MAF >1%). Because the number of clinically diagnosed Alzheimer disease cases in HALST was limited, cohort-specific GWAS estimates would have been underpowered and unstable. Therefore, we used external summary statistics from a large Alzheimer disease GWAS meta-analysis.^35^ To isolate non-*APOE* PRS, variants rs429358 and rs7412—along with 59 variants in linkage disequilibrium (*r*^2^ > 0.025)—were removed, yielding PRS_ADnapoe (with 682 473 SNVs). PRS_ADnapoe scores were analyzed as continuous or in tertiles.

### Statistical Analysis

The major data analyses were conducted from August to December 2025. All analyses were 2-sided, with significance defined as *P* < .05. Group comparisons used analysis of variance for continuous variables and χ^2^ or Fisher exact tests for categorical variables. Mixed-effects models were used to disentangle cross-sectional from longitudinal age-related association with MMSE scores. Covariates included sex, years of education, smoking status, and the first 4 genetic PCs.^12,36^ Smoking status was adjusted for because of its established associations with vascular and neurodegenerative risk factors.^6^ All participants with at least 1 wave of MMSE data were included in the analysis. Following the modeling approach of Caselli et al,^12^ the *j*^th^ MMSE score for the *i*^th^ participant, *Y_ij_* was expressed as follows:

*E*(*Y_ij_*|*B_1i_*) = β_1_ + β_2_Carrier*_i_* + β_3_Age*_i_*_1_ + (β_4_Carrier*_i_* × Age*_i_*_1_) + β_5_Age Squared*_i_*_1_ + (β_6_Carrier*_i_* × Age Squared*_i_*_1_) + β_7_Age*_ij_* + (β_8_Carrier*_i_* × Age*_i_*_j_) + β_9_Age Squared*_ij_* + (β_10_Carrier*_i_* × Age Squared*_ij_*) + β_11_Sex*_i_* + β_12_Education*_i_* + β_13_Smoking*_i_* + β_14_PC1*_i_* + β_15_PC2*_i_* + β_16_PC3*_i_* + β_17_PC4*_i_* + *b*1*_i_*,

where Carrier*_i_* indicates *APOE *ε4 carrier status for participant *i* (1 = carrier; 0 = noncarrier); Age*_ij_* is centered age (age minus 68.2 years) at observation *j*; Sex*_i_* is sex (1 = male; 2 = female); Education*_i_* is total years of formal education; Smoking*_i_* is current smoking status (1 = current smoker; 0 = nonsmoker or former smoker); PC1*_i_* to PC4*_i_* are the top 4 genetic PCs; and* b*_1_*ᵢ* is an individual-specific random intercept.

Age was centered to reduce multicollinearity and improve interpretability. Quadratic age terms were included to capture potential acceleration of cognitive decline in later life. Cross-sectional (between-individual) and longitudinal (within-individual) age components were modeled separately, and effect modification by *APOE* ε4 carrier status was evaluated for both components; the Carrier × Age Squared term (β_10_) served as the primary indicator of accelerated decline.

Subsequent mixed-effects models replaced Carrier*_i_* with (1) *APOE* genotypes: 5 indicator variables (ε2/ε3, ε2/ε4, ε3/ε4, ε2/ε2, ε4/ε4) compared with ε3ε3; (2) *APOE *ε4 dosage: indicator variables for heterozygotes and homozygotes compared with noncarriers; (3) PRS_ADnapoe tertiles: indicators comparing tertile 2 and tertile 3 against tertile 1; (4) PRS_ADnapoe in continuous form; and (5) the interaction between *APOE *ε4 carriage and PRS_ADnapoe (continuous). The potential additive association between *APOE* ε4 carriage and PRS_ADnapoe was evaluated using likelihood ratio tests comparing a full model, including interaction terms between *APOE *ε4 carriage and PRS_ADnapoe, with a reduced model excluding these interaction terms.

Sensitivity analyses for *APOE *ε4 carrier and *APOE *ε4 dosage were performed under various scenarios, including (1) restricting models to participants with complete 2-wave MMSE data (n = 3259); (2) further restricting to those without any missing MMSE items at both waves (n = 2468); (3) excluding participants with baseline MMSE score of less than 21 (n = 4028); (4) excluding those with baseline MMSE score of less than 24 (n = 3590); and (5) applying inverse probability of censoring weighting (IPCW). IPCW accounted for differential attrition. For PRS_ADnapoe, sensitivity analyses were performed among ε3 homozygotes to minimize potential confounding by *APOE *ε4.

All mixed-effects models were fit by restricted maximum likelihood using the PROC MIXED procedure in SAS version 9.4 (SAS Institute). All visualizations were generated using the ggplot2 package in R version 4.5.1 (R Project for Statistical Computing).

## Results

### Participant Characteristics and Genetic Distribution

Among 4392 participants (mean [SD] age, 68.2 [7.8] years; 2359 [53.7%] women), 3636 (82.8%) were noncarriers, 723 (16.5%) were *APOE* ε4 heterozygotes, and 33 (0.8%) were *APOE* ε4 homozygotes, with an ε4 allele frequency of 9.0%. When further stratified by *APOE* genotypes (eFigure 2 in Supplement 1), the ε3/ε3 genotype was the most prevalent (3082 [70.2%]), whereas ε4/ε4 (33 [0.8%]) and ε2/ε2 (26 [0.6%]) were rare. Corresponding ε2 and ε3 allele frequencies were 7.2% and 83.8%. The distribution of PRS_ADnapoe was approximately even (eFigure 3 in Supplement 1).

As shown in Table 1, most sociodemographic and comorbidity characteristics were similar across *APOE *ε4 groups. However, ε4 homozygotes were more likely to be female and less likely to be current smokers. Participants in the highest PRS_ADnapoe tertile (tertile 3) were slightly younger than those in lower tertiles.

**Table 1.** Baseline Demographic Characteristics and Comorbidities Among Participants by *APOE *ε4 Carrier Status and PRS_ADnapoe Tertile

Characteristic	Total sample, No. (%) (n = 4392)	*APOE* ε4 carrier status				PRS_ADnapoe tertile groups			
		Participants, No. (%)			*P* value	Participants, No. (%)			*P* value
		Noncarrier (n = 3636)	Heterozygote (n = 723)	Homozygote (n = 33)		1 (n = 1464)	2 (n = 1464)	3 (n = 1464)	
Age, y									
Mean (SD)	68.2 (7.8)	68.3 (7.8)	68.0 (7.8)	67.7 (7.2)	.71	68.5 (7.9)	68.3 (7.8)	67.8 (7.6)	.02
55-59	835 (19.0)	678 (18.6)	151 (20.9)	6 (18.2)	.82	268 (18.3)	269 (18.4)	298 (20.4)	.03
60-64	692 (15.8)	583 (16.0)	102 (14.1)	7 (21.2)		241 (16.5)	222 (15.2)	229 (15.6)	
65-69	1086 (24.7)	893 (24.6)	185 (25.6)	8 (24.2)		335 (22.9)	383 (26.2)	368 (25.1)	
70-74	943 (21.5)	789 (21.7)	147 (20.3)	7 (21.2)		310 (21.2)	311 (21.2)	322 (22.0)	
75-79	488 (11.1)	405 (11.1)	78 (10.8)	5 (15.2)		178 (12.2)	153 (10.5)	157 (10.7)	
80-84	259 (5.9)	211 (5.8)	48 (6.6)	0		94 (6.4)	98 (6.7)	67 (4.6)	
85-89	70 (1.6)	60 (1.7)	10 (1.4)	0		32 (2.2)	19 (1.3)	19 (1.3)	
90-94	15 (0.3)	14 (0.4)	1 (0.1)	0		6 (0.4)	8 (0.5)	1 (0.1)	
95-99	4 (0.1)	3 (0.1)	1 (0.1)	0		0	1 (0.1)	3 (0.2)	
Educational attainment, mean (SD), y	8.2 (4.8)	8.1 (4.8)	8.4 (4.7)	8.6 (4.5)	.33	8.2 (4.7)	8.2 (4.8)	8.2 (4.8)	>.99
Body mass index, mean (SD)^a^	24.6 (3.5)	24.6 (3.5)	24.6 (3.6)	24.6 (4.2)	.98	24.5 (3.4)	24.7 (3.7)	24.6 (3.4)	.61
Sex									
Male	2033 (46.3)	1697 (46.7)	328 (45.4)	8 (24.2)	.03	687 (46.9)	669 (45.7)	677 (46.2)	.80
Female	2359 (53.7)	1939 (53.3)	395 (54.6)	25 (75.8)		777 (53.1)	795 (54.3)	787 (53.8)	
Marital status									
Married	3285 (74.8)	2732 (75.1)	528 (73.0)	25 (75.8)	.49	1099 (75.1)	1092 (74.6)	1094 (74.7)	.95
Divorced, widowed, or never married	1107 (25.2)	904 (24.9)	195 (27.0)	8 (24.2)		365 (24.9)	372 (25.4)	370 (25.3)	
Smoking status									
Nonsmoker	3155 (71.8)	2603 (71.6)	524 (72.5)	28 (84.8)	.03^b^	1050 (71.7)	1057 (72.2)	1048 (71.6)	.87
Former smoker	650 (14.8)	528 (14.5)	117 (16.2)	5 (15.2)		226 (15.4)	208 (14.2)	216 (14.8)	
Current smoker	587 (13.4)	505 (13.9)	82 (11.3)	0		188 (12.8)	199 (13.6)	200 (13.7)	
Illiteracy									
Yes	928 (21.2)	778 (21.4)	146 (20.2)	4 (12.1)	.34	306 (20.9)	303 (20.7)	319 (21.8)	.74
No	3464 (78.9)	2858 (78.6)	577 (79.8)	29 (87.9)		1158 (79.1)	1161 (79.3)	1145 (78.2)	
Engaged in physical activity									
Yes	3205 (73.0)	2634 (72.4)	546 (75.5)	25 (75.8)	.22	1081 (73.8)	1041 (71.1)	1083 (74.0)	.14
No	1187 (27.0)	1002 (27.6)	177 (24.5)	8 (24.2)		383 (26.2)	423 (28.9)	381 (26.0)	
Diabetes									
Yes	839 (19.1)	703 (19.3)	131 (18.1)	5 (15.2)	.63	260 (17.8)	297 (20.3)	282 (19.3)	.22
No	3553 (80.9)	2933 (80.7)	592 (81.9)	28 (84.8)		1204 (82.2)	1167 (79.7)	1182 (80.7)	
Heart disease									
Yes	916 (20.9)	764 (21.0)	147 (20.3)	5 (15.2)	.66	319 (21.8)	289 (19.7)	308 (21.0)	.39
No	3476 (79.1)	2872 (79.0)	576 (79.7)	28 (84.8)		1145 (78.2)	1175 (80.3)	1156 (79.0)	
Stroke									
Yes	229 (5.2)	193 (5.3)	36 (5.0)	0	.54^b^	88 (6.0)	60 (4.1)	81 (5.5)	.05
No	4163 (94.8)	3443 (94.7)	687 (95.0)	33 (100.0)		1376 (94.0)	1404 (95.9)	1383 (94.5)	

Abbreviation: PRS_ADnapoe, polygenic risk score for Alzheimer disease without *APOE*.

^a^ Body mass index is calculated as weight in kilograms divided by height in meters squared.

^b^ Using Fisher exact test.

Participants excluded at baseline, compared with those included, were older; were more likely to be male and unmarried; had lower educational attainment and higher rates of illiteracy; were more often current or former smokers; were less physically active; and had lower cognitive performance, whereas comorbidities were comparable between groups (eTable 1 in Supplement 1). In addition, participants with only baseline MMSE data, compared with those with complete 2-wave MMSE data, demonstrated similar differences and had higher prevalences of diabetes, heart disease, and stroke.

### MMSE Distributions and Crude Change

Follow-up spanned 4.50 to 11.42 years, with a mean (SD) of 6.26 (0.86) years. MMSE distributions and longitudinal changes are shown in eFigure 4 in Supplement 1. Cross-sectional MMSE distributions were left-skewed, whereas longitudinal changes were more symmetrically distributed.

Table 2 shows the mean (SD) MMSE scores across time points. In the 2-wave subset (n = 3259), mean MMSE declined by 1.3 points (SD, 2.9), and the mean (SD) annual decline was −0.2 (0.5). Stratified by *APOE *ε4 dosage, cross-sectional MMSE means were similar, but longitudinal decline differed. No differences in cross-sectional or longitudinal MMSE were observed across PRS_ADnapoe tertiles. Additional cross-sectional analysis by 5-year age intervals (Figure, A) showed divergence between *APOE* ε4 carriers and noncarriers beginning between ages 70 and 75 years.

**Table 2.** Distribution of MMSE Scores at Different Time Points and the Corresponding Changes in the Subsample With Complete 2-Wave MMSE Scores, by *APOE* Carrier Status and PRS_ADnapoe Tertiles

Factor	Overall, mean (SD)	*APOE* ε4 carrier status					PRS_AD_napoe_ tertile groups				
		Participants, mean (SD)			*P* value	Participants, mean (SD)				*P* value
		Noncarrier	Heterozygotes	Homozygotes		1		2	3	
Total sample, No.	4392	3636	723	33	NA	1464		1464	1464	NA
MMSE at wave 1	26.5 (3.4)	26.5 (3.4)	26.6 (3.4)	27.0 (3.0)	.72	26.5 (3.5)		26.5 (3.5)	26.6 (3.3)	.35
Baseline MMSE–only sample, No.	1133	954	168	11	NA	381		382	370	NA
MMSE at Wave1	25.0 (3.9)	25.0 (3.9)	25.0 (3.9)	27.0 (2.9)	.24	25.0 (3.9)		24.9 (4.0)	25.2 (3.8)	.62
Complete 2-wave sample, No.	3259	2682	555	22	NA	1083		1082	1094	NA
Cross-sectional										
MMSE at wave 1	27.1 (3.0)	27.1 (3.0)	27.0 (3.1)	27.0 (3.1)	.94	27.0 (3.1)		27.0 (3.1)	27.1 (2.9)	.61
MMSE at wave 2	25.7 (4.1)	25.8 (4.0)	25.5 (4.4)	24.4 (6.4)	.11	25.7 (3.9)		25.7 (4.3)	25.8 (4.0)	.84
Longitudinal										
Change in MMSE	−1.3 (2.9)	−1.3 (2.8)	−1.5 (3.0)	−2.6 (4.6)	.03	−1.3 (2.6)		−1.4 (3.1)	−1.4 (2.9)	.79
Annual change in MMSE	−0.2 (0.5)	−0.2 (0.5)	−0.2 (0.5)	−0.4 (0.8)	.02	−0.2 (0.4)		−0.2 (0.5)	−0.2 (0.5)	.82

Abbreviations: MMSE, Mini Mental State Examination; NA, not applicable; PRS_ADnapoe, polygenic risk score for Alzheimer disease, without *APOE*.

**Figure.**
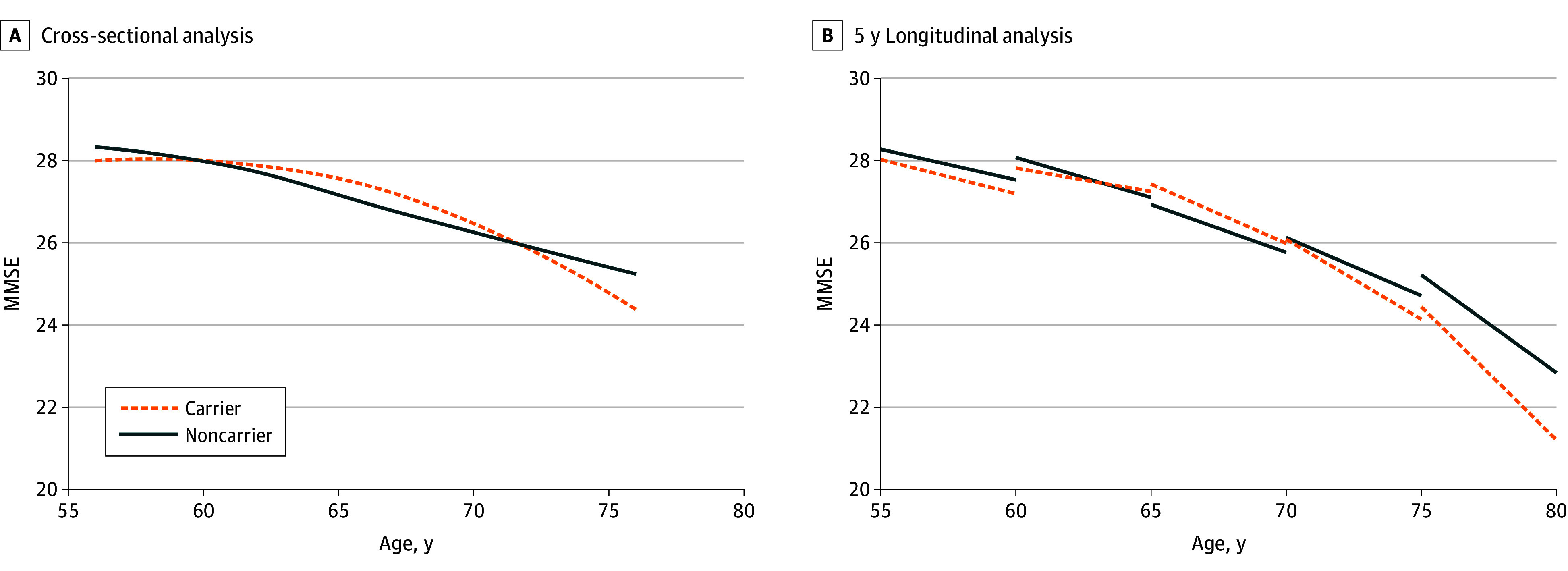
Line Graphs of Cognitive Change Among *APOE *ε4 Carrier and Noncarriers Data were collected among community-dwelling adults aged 55 years and older in Tawain who were recruited from 2009 to 2013. A, The cross-sectional analysis included 4392 participants at wave 1. B, The longitudinal analysis included 3259 participants who completed examinations at 2 waves. MMSE indicates Mini-Mental State Examination.

### Longitudinal Association Between Age and MMSE Decline

Mixed-effects models revealed a significantly stronger quadratic longitudinal association between age and MMSE decline among *APOE* ε4 carriers than noncarriers (estimate, −0.005; SE, 0.001; *P* = .001) (Table 3). In 5-year trajectories (Figure, B), divergence became evident after approximately age 70 years. When modeled by dosage (Table 4), both ε4 heterozygotes (estimate, −0.004; SE, 0.001; *P* = .001) and homozygotes (estimate, −0.017; SE, 0.008; *P* = .03) exhibited greater age-related decline than noncarriers, with the strongest association observed among homozygotes. eFigure 5A and B in Supplement 1 show projected trajectories by ε4 dosage. When the same models were applied to PRS_ADnapoe tertiles, no significant differences were observed in the longitudinal age-related MMSE trajectories, either in the overall sample or among ε3 homozygotes (eTable 2 in Supplement 1). Similar null findings were obtained when PRS_ADnapoe was modeled as a continuous variable (eTable 3 in Supplement 1).

**Table 3.** Mixed-Effects Models of *APOE* ε4 Carriers and Noncarriers

Model parameters	Estimate (SE)	*P* value
Fixed effects		
Intercept	24.060 (0.187)	<.001
Carrier	0.124 (0.142)	.38
Age at wave 1 (centered)	0.078 (0.010)	<.001
Carrier × age at wave 1 (centered)	0.041 (0.023)	.08
Age at wave 1 squared (centered)	0.003 (0.001)	.001
Carrier × age at wave 1 squared (centered)	0.002 (0.002)	.38
Age at wave 2 (centered)	−0.168 (0.009)	<.001
Carrier × age at wave 2 (centered)	−0.027 (0.021)	.20
Age at wave 2 squared (centered)	−0.006 (0.001)	<.001
Carrier × age at wave 2 squared (centered)	−0.005 (0.001)	.001
Sex, female vs male	−0.397 (0.087)	<.001
Educational attainment	0.402 (0.009)	<.001
Smoking, current vs non or former	−0.118 (0.124)	.34
PC1	−26.010 (2.571)	<.001
PC2	7.037 (2.673)	.01
PC3	0.583 (2.567)	.82
PC4	1.700 (2.627)	.52
Random effects		
Intercept	4.023 (0.150)	<.001
Residual	3.932 (0.098)	<.001

Abbreviation: PC, principal component.

**Table 4.** Mixed-Effects Models of *APOE* ε4 Homozygous Persons, Heterozygous Persons, and Noncarriers

Model parameters	Estimate (SE)	*P* value
Fixed effects		
Intercept	24.050 (0.187)	<.001
Heterozygotes	0.129 (0.144)	.37
Homozygotes	0.362 (0.722)	.62
Age at wave 1 (centered)	0.078 (0.010)	<.001
Heterozygotes × age at wave 1 (centered)	0.038 (0.024)	.11
Homozygotes × age at wave 1 (centered)	0.137 (0.109)	.21
Age at wave 1 squared (centered)	0.003 (0.001)	.001
Heterozygotes × age at wave 1 squared (centered)	0.001 (0.002)	.51
Homozygotes × age at wave 1 squared (centered)	0.014 (0.012)	.25
Age at wave 2 (centered)	−0.168 (0.009)	<.001
Heterozygotes × age at wave 2 (centered)	−0.020 (0.021)	.35
Homozygotes × age at wave 2 (centered)	−0.213 (0.095)	.02
Age at wave 2 squared (centered)	−0.006 (0.001)	<.001
Heterozygotes × age at wave 2 squared (centered)	−0.004 (0.001)	.001
Homozygotes × age at wave 2 squared (centered)	−0.017 (0.008)	.03
Sex, female vs male	−0.392 (0.087)	<.001
Educational attainment	0.403 (0.009)	<.001
Smoking, current vs non or former	−0.113 (0.124)	.36
PC1	−26.010 (2.570)	<.001
PC2	7.124 (2.673)	.01
PC3	0.528 (2.568)	.84
PC4	1.773 (2.627)	.50
Random effects		
Intercept	4.022 (0.150)	<.001
Residual	3.928 (0.098)	<.001

Abbreviation: PC, principal component.

The evaluation of additive associations between *APOE *ε4 carriage and PRS_ADnapoe with longitudinal MMSE change is presented in eTable 4 in Supplement 1. In model 1, *APOE *ε4 carriage and PRS_ADnapoe were included as main effects, whereas model 2 additionally included their interaction term. Both models revealed a significantly stronger quadratic longitudinal association between age and MMSE decline among *APOE *ε4 carriers compared with noncarriers. In contrast, no significant differences were observed across PRS_ADnapoe levels in either model. The likelihood ratio test comparing model 1 and model 2 was not significant, indicating no evidence of an interaction between *APOE* ε4 carrier status and PRS_ADnapoe on cognitive decline.

### Other *APOE* Genotypes and MMSE Scores

Given the putative protective association of *APOE* ε2 and the increased risk associated with *APOE* ε4, we further examined MMSE changes across *APOE* genotypes. No significant differences were observed in cross-sectional MMSE scores, total MMSE change, or annual changes among *APOE* ε2 carriers (excluding ε2/ε4), *APOE* ε4 carriers, and noncarriers (eTable 5 in Supplement 1). To facilitate comparison, cross-sectional and longitudinal MMSE trajectories for these 3 groups are shown in eFigure 6A and B in Supplement 1. The trajectories of *APOE* ε2 carriers did not significantly differ from those of *APOE* ε4 carriers or noncarriers, providing no evidence of a protective association of *APOE* ε2 with cognitive decline as measured by MMSE in this Taiwanese cohort. In mixed-effects models (eTable 6 in Supplement 1), compared with ε3/ε3, only ε3/ε4 and ε4/ε4 showed significant quadratic longitudinal associations with aging. Since *APOE* ε2 carriers did not differ significantly from *APOE* ε4 noncarriers, all *APOE* ε4 noncarriers were pooled in subsequent analyses (Tables 3 and 4).

### Sensitivity Analyses

The results of sensitivity analyses for *APOE *ε4 carriers appear in eTable 7 in Supplement 1. In scenario 1, in which analyses restricted to participants with 2 MMSE waves completed, the association between *APOE* ε4 carrier status and MMSE score remained significant. In scenario 2, which further excluded participants with any item-level missingness in either MMSE wave, the result attenuated and became nonsignificant. In scenarios 3 and 4, which excluded participants with baseline MMSE scores less than 21 and less than 24, respectively, the quadratic longitudinal age-related decline was not statistically significant. In scenario 5, in which the model was adjusted using IPCW, the quadratic longitudinal age-related decline regained statistical significance.

The results of sensitivity analyses for *APOE *ε4 dosage groups are presented in eTable 8 in Supplement 1. In scenario 1, in which analyses were restricted to participants who completed both waves of MMSE, *APOE *ε4 heterozygotes continued to show a significant quadratic age–related decline. The results among ε4 homozygotes were similar, but the finding was not statistically significant. In scenario 2, excluding participants with any item-level missingness in MMSE, none of the age-related interaction terms were statistically significant for either dosage group. In scenario 3, which excluded individuals with baseline MMSE scores of less than 21, the interaction of homozygotes with the quadratic age term was not statistically significant. Scenario 4, excluding those with MMSE scores of less than 24, yielded similar nonsignificant results across both dosage groups. However, in scenario 5, where models were adjusted using IPCW, the quadratic age–associated declines for both *APOE *ε4 heterozygotes and homozygotes were statistically significant.

## Discussion

In this population-based Taiwanese cohort, 16.5% of participants were *APOE *ε4 heterozygotes and 0.8% were *APOE *ε4 homozygotes. In the 2-wave subset, the annual MMSE decline averaged −0.2 points. Annual MMSE decline was greater with higher ε4 dosage, whereas no differences were observed among PRS_ADnapoe tertiles. After adjustment for covariates, *APOE *ε4 carriers (heterozygotes and homozygotes) exhibited significantly stronger quadratic longitudinal associations between age and MMSE decline, while no such association was detected for PRS_ADnapoe. Specifically, ε4 carriers showed accelerated declines in MMSE scores beginning at approximately age 70 years, with the strongest association among ε4 homozygotes.

Our results align with prior longitudinal studies demonstrating *APOE* ε4–associated cognitive decline before overt dementia and extend these findings to an Asian population using a modeling approach that separates cross-sectional from longitudinal aging effects. These findings underscore the consistency of *APOE *ε4’s association with cognitive trajectories across populations, despite ethnic differences in allele frequency.^16,18,37^ These findings also echo prior research indicating that a higher *APOE* ε4 allele load is associated with alterations in brain morphology.^38^

The prevalence of *APOE *ε4 heterozygotes and homozygotes in our study was very similar to that reported in a cognitively normal Japanese cohort (n = 1700; 17.2% heterozygotes and 0.8% homozygotes).^39^ Our estimates were slightly lower than those from a memory clinic cohort in northern Taiwan (21.2% heterozygotes and 0.9% homozygotes).^40^ In general, these estimates indicate that the Taiwanese population is at the lower end of *APOE *ε4 allele frequency globally.^39,41^

To our knowledge, this is the first study in a Chinese population to demonstrate *APOE*–associated acceleration of MMSE decline among cognitively healthy adults in midlife and old age. Using mixed-effects modeling that accounts for both cross-sectional and longitudinal aging effects, *APOE *ε4 carriage was associated with cognitive decline over time.

Previous studies, primarily in US and UK cohorts, often used a battery of cognitive tests, with accelerated decline observed only in specific domains.^12,13,14,15,17,20,21^ One study that included MMSE in the test battery detected a quadratic aging effect in global cognition but not in MMSE.^12^ Two factors may explain this discrepancy. First, our study included only adults aged 55 years and older, whereas the previous study included participants aged 21 to 97 years, potentially diluting the longitudinal effect. Second, our sample size was substantially larger, improving power to detect group differences.

The absence of a detectable association between PRS_ADnapoe and cognitive decline may reflect the relatively younger age of our participants and the limited duration of follow-up. A prior US study found that associations between non-*APOE* PRS and cognition tend to emerge 5 to 15 years later than associations involving *APOE *ε4.^20^ Since *APOE *ε4 carriers in our cohort showed accelerated decline beginning around age 70 years, PRS_ADnapoe associations may require longer observation periods to become apparent. Furthermore, the adverse associations between PRS and cognitive outcomes are often stronger among *APOE *ε4 carriers, suggesting a potential interaction.^21^ Although interaction terms were not significant here, investigating such interactions in Asian populations remains an important future direction.

Despite *APOE* ε2 being associated with reduced AD risk in previous studies,^11,37^ we did not detect a clear protective association of *APOE* ε2 with MMSE trajectories in this cohort. Several factors may contribute to this null finding, including the relatively modest proportion of *APOE* ε2 carriers and the use of MMSE as a global rather than domain-specific measure. Larger studies with more sensitive cognitive batteries will be needed to clarify the role of *APOE* ε2 on cognitive aging in Taiwanese populations.

*APOE* ε4 has been suggested to confer cognitive advantages earlier in life, raising the possibility of antagonistic pleiotropy.^42^ In our cohort, MMSE trajectories for ε4 carriers and noncarriers were similar throughout their 50s and 60s, with divergence emerging only around age 70 years. This pattern does not provide clear evidence of early cognitive advantages among ε4 carriers but is broadly consistent with the notion that ε4-related disadvantages become more apparent with advancing age.

Our findings support consideration of *APOE *ε4 testing and targeted risk communication in midlife, given that accelerated MMSE decline among ε4 carriers begins at approximately age 70 years. Approximately 17.3% of our participants were ε4 carriers who might benefit from early counseling and preventive strategies. Several interventions—such as Mediterranean-style diets,^43^ structured cognitive training,^44^ and regular physical activity^45^—show promise in attenuating cognitive decline before dementia onset. Future research should evaluate the cost-effectiveness of these interventions, define optimal timing and intensity, explore interactions with broader polygenic risk, examine outcomes beyond MMSE, and extend follow-up durations.

### Limitations

This study has several limitations. First, cognitive change was assessed only with the MMSE at 2 time points, and MMSE performance is influenced by educational level and illiteracy. Although we adjusted for years of education and used within-person change, the MMSE is a global screening tool with limited sensitivity to subtle or early cognitive decline. This limitation likely led to underestimation of true cognitive change and may partly explain why divergence between *APOE* ε4 carriers and noncarriers became apparent only around age 70 years. More sensitive, domain-specific assessments may detect earlier and larger genotype-related differences. Second, missing genetic data and attrition during follow-up may have introduced selection bias, although results were robust in weighted analyses. Third, we lacked concurrent AD biomarker data (eg, amyloid or plasma phosphorylated tau [pTau], including pTau217) at the time of this analysis and therefore could not assess whether *APOE*-associated differences in MMSE trajectories were mediated by AD neuropathological changes or remained independent of such pathology. Fourth, PRS_ADnapoe scores were derived primarily from European-ancestry GWAS, which may reduce their performance in this population. Finally, as only 84 participants met the proxy criterion for dementia (defined as a wave-2 MMSE score <16), the statistical power was insufficient to evaluate the association between *APOE* genotypes and incident dementia.

## Conclusions

In this Taiwanese community cohort study with relatively low educational attainment, *APOE* ε4 carriers exhibited accelerated age-related cognitive decline that was detectable with the MMSE around age 70 years. The association scaled with *APOE* ε4 dosage and remained robust in sensitivity analyses. In contrast, PRS_ADnapoe scores were not associated with MMSE decline over the study period. These findings support early risk awareness and lifestyle interventions for *APOE* ε4 carriers, while highlighting the need for longer follow-up to clarify the contribution of non-*APOE* polygenic risk.

## Notes

Supplement 1. **eFigure 1.** Summary of Censoring and Causes of Attrition in the Study Process **eFigure 2.** Distribution of the APOE Genotype Among 4392 Participants in HALST **eFigure 3.** Distribution of the PRS for AD Not Containing APOE ε4 in 4392 Participants in HALST **eFigure 4.** MMSE Scores Among Community-Dwellers Aged 55 Years or Older and Recruited From 2009 to 2013 in Taiwan **eFigure 5.** Cross-Sectional and Longitudinal Analysis for Projected MMSE Trajectories Among APOE ε4 Homozygotes, APOE ε4 Heterozygotes, and Noncarriers **eFigure 6.** Cross-Sectional and Longitudinal Analysis for Among APOE ε2 Carriers (excluding ε2/ε4), APOE ε4 Carriers, and ε3/ε3 Carriers **eTable 1.** Comparison of Demographic Characteristics and Comorbidities Among Participants Included or Excluded at Baseline and Those With or Without Complete 2-Wave MMSE During Follow-Up **eTable 2.** Mixed Models of PRS_ADnapoe Tertiles Among Community-Dwellers Aged 55 Years or Older and Recruited From 2009 to 2013 in Taiwan **eTable 3.** Mixed Models of PRS_ADnapoe as Continuous Variable Among Community-Dwellers Aged 55 Years or Older and Recruited From 2009 to 2013 in Taiwan **eTable 4.** Examination of the Additive Effect Between APOE ε4 Carriage and PRS_ADnapoe via Likelihood Ratio Test Between 2 Models Among Community-Dwellers Aged 55 Years or Older and Recruited From 2009 to 2013 in Taiwan **eTable 5.** Distribution of MMSE Scores Across the Total Sample, Participants With 1-Wave MMSE Only, and Participants With Complete 2-Wave MMSE **eTable 6.** Mixed Models of Different APOE Genotypes Among Community-Dwellers Aged 55 Years or Older and Recruited From 2009 to 2013 in Taiwan **eTable 7.** Sensitivity Analysis for Association Between APOE ε4 Carriers and Noncarriers and MMSE Score Change in Different Scenarios **eTable 8.** Sensitivity Analysis for Association Between APOE ε4 Homozygous, Heterozygous, and Noncarrier Status and MMSE Score Change in Different Scenarios

Supplement 2.
Data Sharing Statement
