# Supplementary material for: APOE ε4 and Accelerated Cognitive Decline Among Cognitively Healthy Middle-Aged and Older Adults
Source: JAMA Netw Open. 2026 Mar 6;9(3):e260853. doi: 10.1001/jamanetworkopen.2026.0853 (PMC12966930; doi:10.1001/jamanetworkopen.2026.0853)
Supplement: Supplement 1. — eFigure 1. Summary of Censoring and Causes of Attrition in the Study Process eFigure 2. Distribution of the APOE Genotype Among 4392 Participants in HALST eFigure 3. Distribution of the PRS for AD Not Containing APOE ε4 in 4392 Participants in HALST eFigure 4. MMSE Scores Among Community-Dwellers Aged 55 Years or Older and Recruited From 2009 to 2013 in Taiwan eFigure 5. Cross-Sectional and Longitudinal Analysis for Projected MMSE Trajectories Among APOE ε4 Homozygotes, APOE ε4 Heterozygotes, and Noncarriers eFigure 6. Cross-Sectional and Longitudinal Analysis for Among APOE ε2 Carriers (excluding ε2/ε4), APOE ε4 Carriers, and ε3/ε3 Carriers eTable 1. Comparison of Demographic Characteristics and Comorbidities Among Participants Included or Excluded at Baseline and Those With or Without Complete 2-Wave MMSE During Follow-Up eTable 2. Mixed Models of PRS_ADnapoe Tertiles Among Community-Dwellers Aged 55 Years or Older and Recruited From 2009 to 2013 in Taiwan eTable 3. Mixed Models of PRS_ADnapoe as Continuous Variable Among Community-Dwellers Aged 55 Years or Older and Recruited From 2009 to 2013 in Taiwan eTable 4. Examination of the Additive Effect Between APOE ε4 Carriage and PRS_ADnapoe via Likelihood Ratio Test Between 2 Models Among Community-Dwellers Aged 55 Years or Older and Recruited From 2009 to 2013 in Taiwan eTable 5. Distribution of MMSE Scores Across the Total Sample, Participants With 1-Wave MMSE Only, and Participants With Complete 2-Wave MMSE eTable 6. Mixed Models of Different APOE Genotypes Among Community-Dwellers Aged 55 Years or Older and Recruited From 2009 to 2013 in Taiwan eTable 7. Sensitivity Analysis for Association Between APOE ε4 Carriers and Noncarriers and MMSE Score Change in Different Scenarios eTable 8. Sensitivity Analysis for Association Between APOE ε4 Homozygous, Heterozygous, and Noncarrier Status and MMSE Score Change in Different Scenarios [file jamanetwopen-e260853-s001.pdf]

Supplemental Online Content

Chung YE, Chung R, Hsu C, et al. *APOE* ε4 and accelerated cognitive decline among cognitively healthy middle-aged and older adults. *JAMA Netw Open.* 2026;9(3):e260853. doi:10.1001/jamanetworkopen.2026.0853

eFigure 1. Summary of Censoring and Causes of Attrition in the Study Process

eFigure 2. Distribution of the APOE Genotype Among 4392 Participants in HALST

eFigure 3. Distribution of the PRS for AD Not Containing APOE ε4 in 4392 Participants in HALST

eFigure 4. MMSE Scores Among Community-Dwellers Aged 55 Years or Older and Recruited From 2009 to 2013 in Taiwan

eFigure 5. Cross-Sectional and Longitudinal Analysis for Projected MMSE Trajectories Among APOE ε4 Homozygotes, APOE ε4 Heterozygotes, and Noncarriers

eFigure 6. Cross-Sectional and Longitudinal Analysis for Among APOE ε2 Carriers (excluding ε2/ε4), APOE ε4 Carriers, and ε3/ε3 Carriers

eTable 1. Comparison of Demographic Characteristics and Comorbidities Among Participants Included or Excluded at Baseline and Those With or Without Complete 2-Wave MMSE During Follow-Up

eTable 2. Mixed Models of PRS_ADnapoe Tertiles Among Community-Dwellers Aged 55 Years or Older and Recruited From 2009 to 2013 in Taiwan

eTable 3. Mixed Models of PRS_ADnapoe as Continuous Variable Among Community-Dwellers Aged 55 Years or Older and Recruited From 2009 to 2013 in Taiwan

eTable 4. Examination of the Additive Effect Between APOE ε4 Carriage and PRS_ADnapoe via Likelihood Ratio Test Between 2 Models Among Community-Dwellers Aged 55 Years or Older and Recruited From 2009 to 2013 in Taiwan

eTable 5. Distribution of MMSE Scores Across the Total Sample, Participants With 1-Wave MMSE Only, and Participants With Complete 2-Wave MMSE

© 2026 Chung YCE et al. *JAMA Network Open*.

eTable 6. Mixed Models of Different APOE Genotypes Among Community-Dwellers Aged 55 Years or Older and Recruited From 2009 to 2013 in Taiwan

eTable 7. Sensitivity Analysis for Association Between APOE ε4 Carriers and Noncarriers and MMSE Score Change in Different Scenarios

eTable 8. Sensitivity Analysis for Association Between APOE ε4 Homozygous, Heterozygous, and Noncarrier Status and MMSE Score Change in Different Scenarios

This supplemental material has been provided by the authors to give readers additional information about their work.

© 2026 Chung YCE et al. *JAMA Network Open*.

eFigure 1. Summary of Censoring and Causes of Attrition in the Study Process

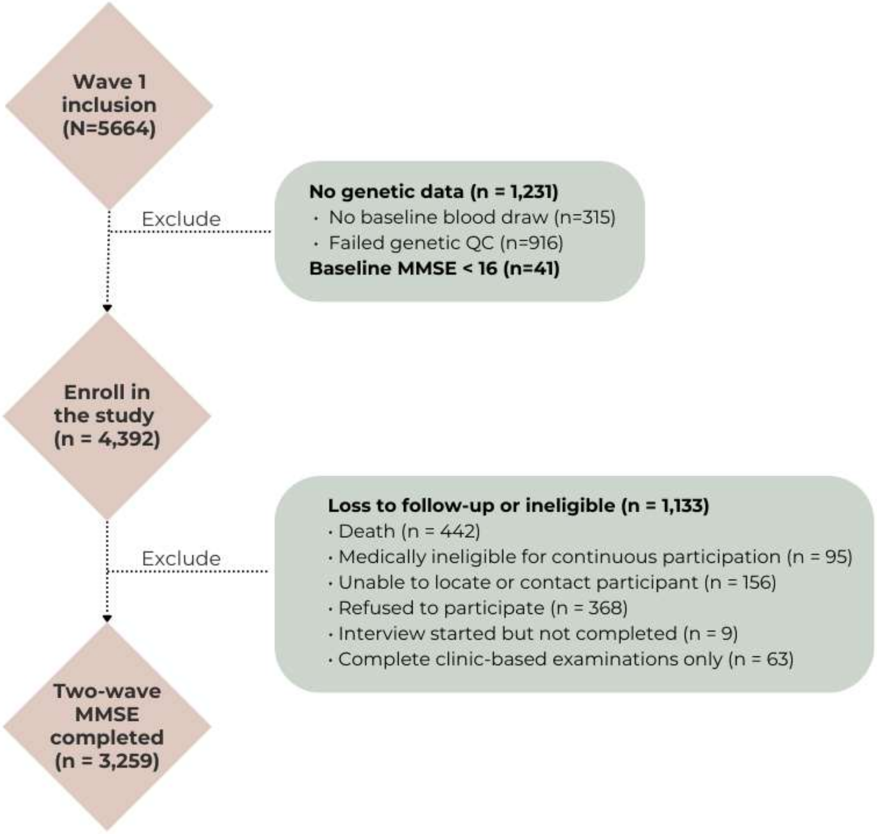

© 2026 Chung YCE et al. *JAMA Network Open*.

eFigure 2. Distribution of the *APOE* Genotype Among 4392 Participants in HALST

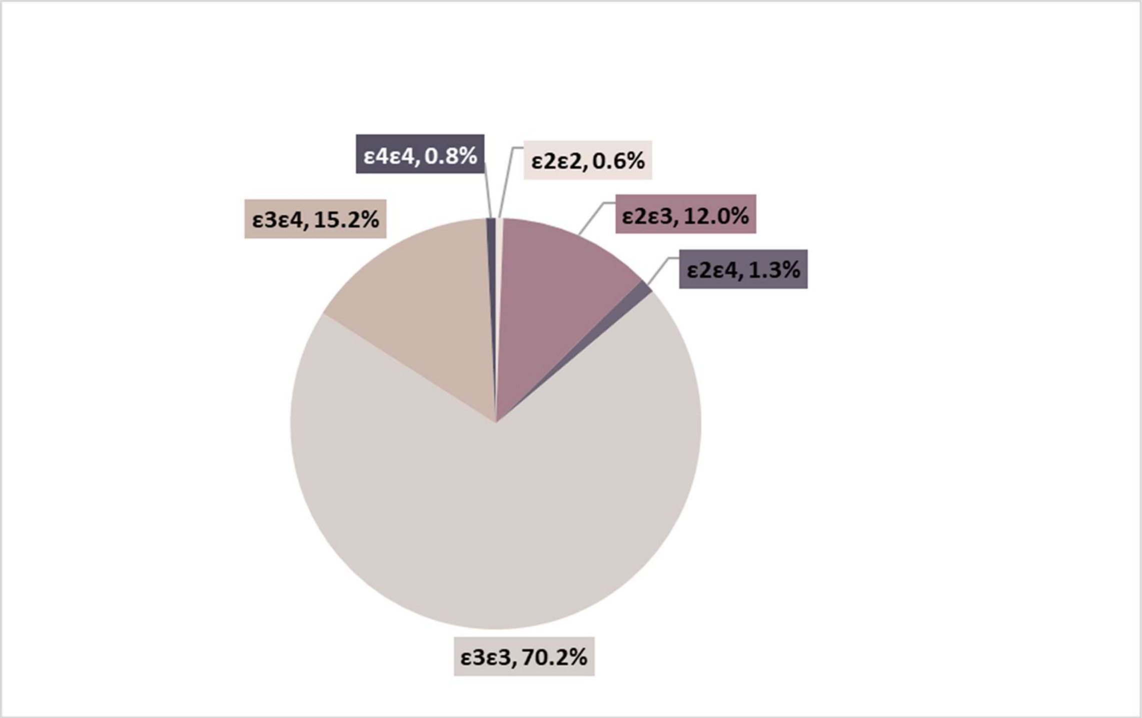

© 2026 Chung YCE et al. *JAMA Network Open*.

eFigure 3. Distribution of the PRS for AD Not Containing *APOE* ε4 in 4392 Participants in HALST

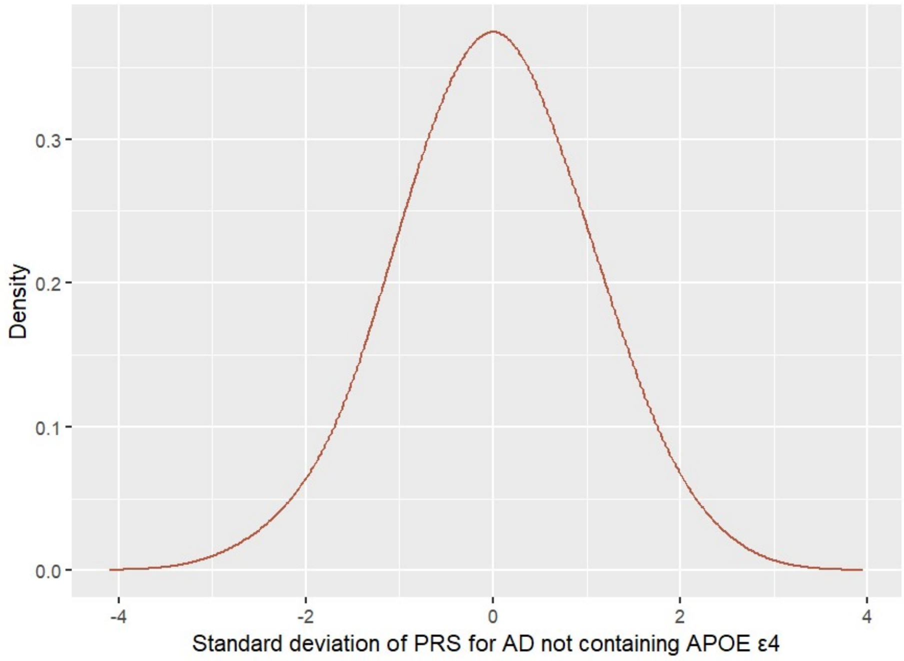

© 2026 Chung YCE et al. *JAMA Network Open*.

eFigure 4. MMSE Scores Among Community-Dwellers Aged 55 Years or Older and Recruited From 2009 to 2013 in Taiwan

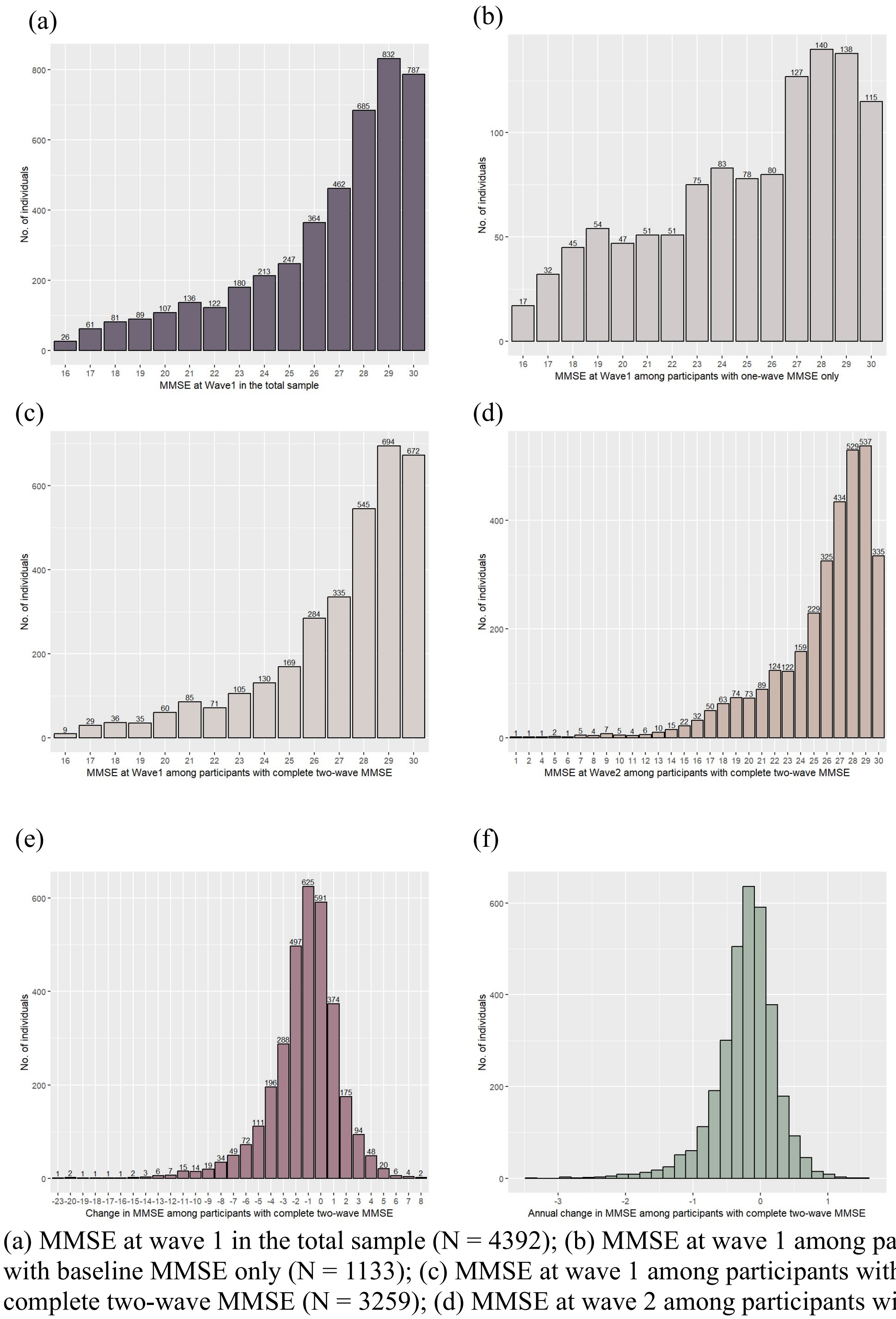

(a) MMSE at wave 1 in the total sample (N = 4392); (b) MMSE at wave 1 among participants with baseline MMSE only (N = 1133); (c) MMSE at wave 1 among participants with complete two-wave MMSE (N = 3259); (d) MMSE at wave 2 among participants with complete two-wave MMSE (N = 3259); (e) Change in MMSE among participants with complete two-wave MMSE (N = 3259); (f) Annual change in MMSE among participants with complete two-wave MMSE (N = 3259).

© 2026 Chung YCE et al. *JAMA Network Open*.

eFigure 5. Cross-Sectional and Longitudinal Analysis for Projected MMSE Trajectories Among *APOE* ε4 Homozygotes, *APOE* ε4 Heterozygotes, and Noncarriers

(a)

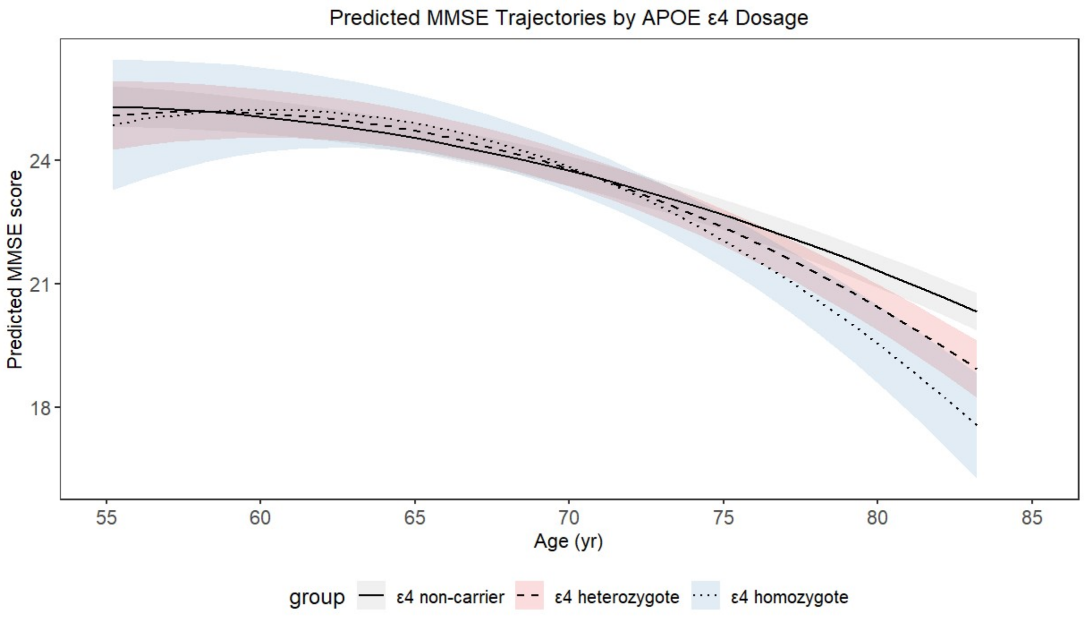

(b)

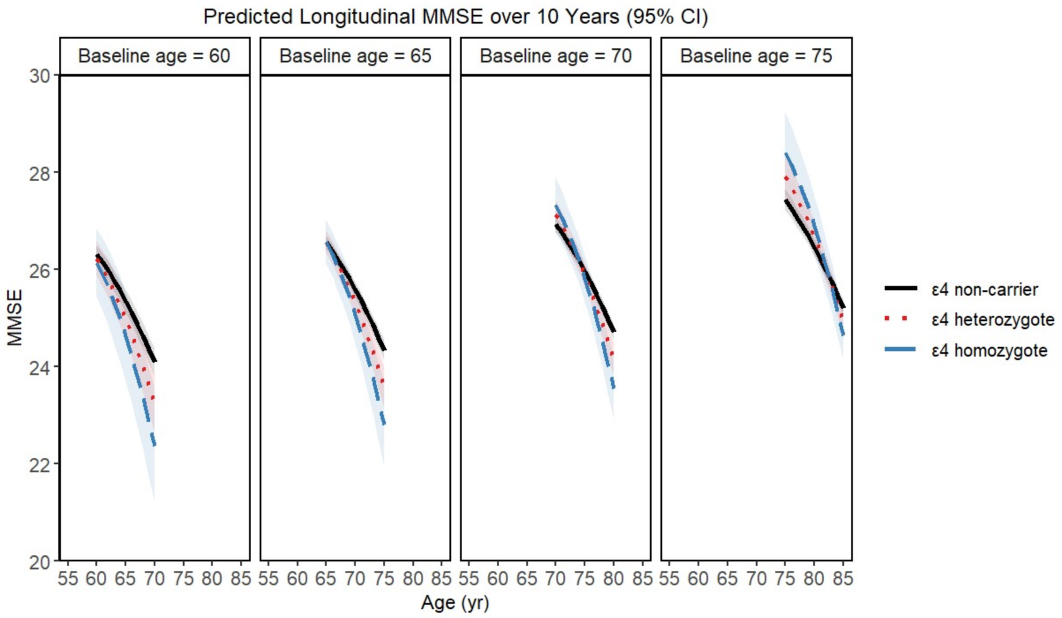

A, Cross-sectional analysis among community-dwellers aged ≥55 years and recruited from 2009 to 2013 in Taiwan (N = 4392); B, 5-year longitudinal analysis among community-dwellers aged ≥55 years and recruited from 2009 to 2013 in Taiwan (N = 3259).

© 2026 Chung YCE et al. *JAMA Network Open*.

eFigure 6. Cross-Sectional and Longitudinal Analysis for Among *APOE* ε2 Carriers (excluding ε2/ε4), *APOE* ε4 Carriers, and ε3/ε3 Carriers

(a)

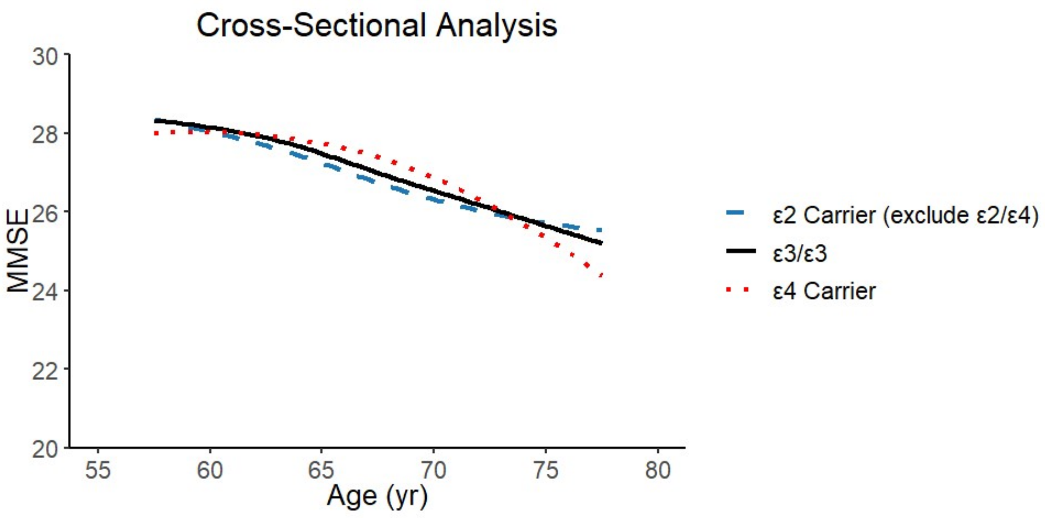

(b)

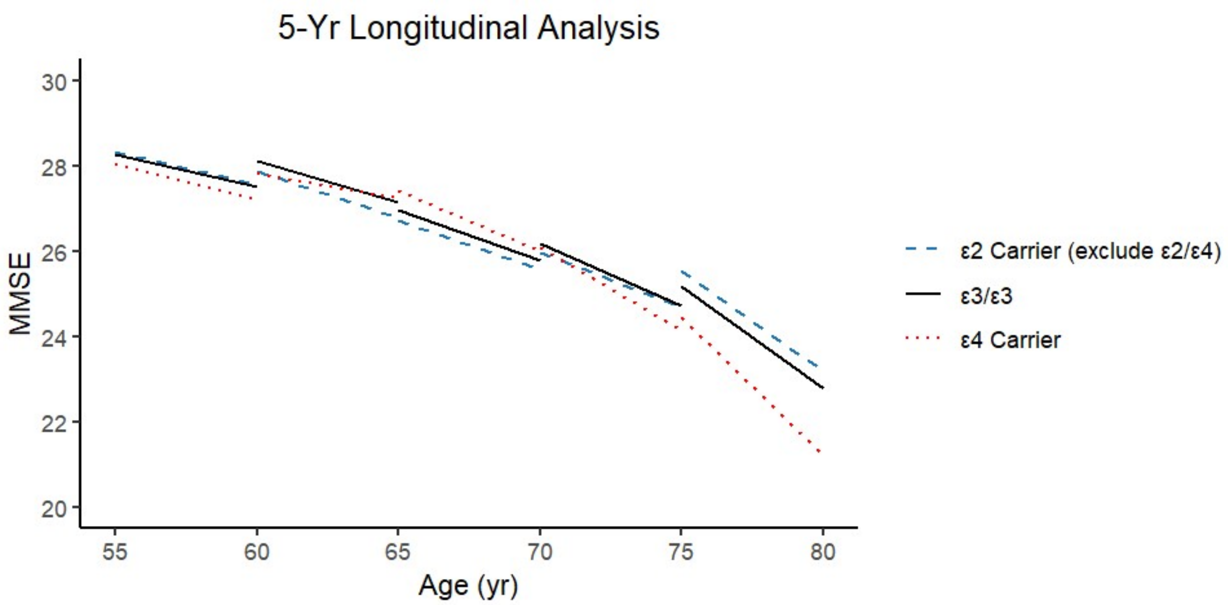

Figure S6. The (a) Cross-sectional analysis for cognitive change among *APOE ε2* carrier (exclude ε2/ε4), *APOE ε4* carrier, and ε3/ε3 among community-dwellers aged ≥55 years and recruited from 2009 to 2013 in Taiwan (N = 4392); (b) Five year longitudinal analysis for cognitive change among *APOE ε2* carrier (exclude ε2/ε4), *APOE ε4* carrier, and ε3/ε3 among community-dwellers aged ≥55 years and recruited from 2009 to 2013 in Taiwan (N = 3259).

© 2026 Chung YCE et al. *JAMA Network Open*.

eTable 1. Comparison of Demographic Characteristics and Comorbidities Among Participants Included or Excluded at Baseline and Those With or Without Complete 2-Wave MMSE During Follow-Up

Variables	Baseline			Follow-up		
	Excluded (n=1271)	Included (n=4392)	P-value	Baseline MMSE only (n=1133)	With two-wave MMSE (n=3259)	P-value
Age, mean (SD)	74.4 (8.0)	68.2 (7.8)	**<0.001**	72.3 (8.3)	66.8 (7.1)	**<0.001**
Educational year, mean (SD)	6.4 (4.8)	8.2 (4.8)	**<0.001**	6.6 (4.7)	8.7 (4.7)	**<0.001**
MMSE at Wave1, mean (SD)	24.7 (4.5)	26.5 (3.4)	**<0.001**	25.0 (3.9)	27.1 (3.0)	**<0.001**
Sex, n (%)						**0.04**
Male	642 (50.5)	2033 (46.3)	**0.01**	554 (48.9)	1479 (45.4)	
Female	629 (49.5)	2359 (53.7)		579 (51.1)	1780 (54.6)	
Marital status, n (%)			**<0.001**			**<0.001**
Married	873 (68.7)	3285 (74.8)		766 (67.6)	2519 (77.3)	
Divorced/widowed/never married	398 (31.3)	1107 (25.2)		367 (32.4)	740 (22.7)	
Smoking status, n (%)			**<0.001**			**<0.001**
Non-smoker	891 (70.1)	3155 (71.8)		737 (65.0)	2418 (74.2)	
Former smoker	244 (19.2)	650 (14.8)		194 (17.1)	456 (14.0)	
Current smoker	136 (10.7)	587 (13.4)		202 (17.8)	385 (11.8)	
Illiteracy, n (%)			**<0.001**			**<0.001**
Yes	472 (37.1)	928 (21.2)		414 (36.5)	514 (15.8)	
No	799 (62.9)	3464 (78.9)		719 (63.5)	2745 (84.2)	
Engaged in physical activity, n (%)			**<0.001**			**<0.001**
Yes	834 (65.6)	3205 (73.0)		752 (66.4)	2453 (75.3)	
No	437 (34.4)	1187 (27.0)		381 (33.6)	806 (24.7)	

© 2026 Chung YCE et al. *JAMA Network Open*.

Diabetes, n (%)			0.06			**<0.001**
Yes	212 (16.7)	839 (19.1)		278 (24.5)	561 (17.2)	
No	1059 (83.3)	3553 (80.9)		855 (75.5)	2698 (82.8)	
Heart disease, n (%)			0.05			**<0.001**
Yes	299 (23.5)	916 (20.9)		294 (25.9)	622 (19.1)	
No	972 (76.5)	3476 (79.1)		839 (74.1)	2637 (80.9)	
Stroke, n (%)			0.44			**<0.001**
Yes	74 (5.8)	229 (5.2)		109 (9.6)	120 (3.7)	
No	1197 (94.2)	4163 (94.8)		1024 (90.4)	3139 (96.3)	

© 2026 Chung YCE et al. *JAMA Network Open*.

eTable 2. Mixed Models of PRS_ADnapoe Tertiles Among Community-Dwellers Aged 55 Years or Older and Recruited From 2009 to 2013 in Taiwan (N = 4392)

	Overall (N = 4392)			Among participants with ε3 homozygotes (n=3082)		
Model parameters	Estimate	SE	P	Estimate	SE	P
Fixed effects						
Intercept	**24.100**	**0.201**	**<0.001**	**24.270**	**0.263**	**<0.001**
$PRS\_AD_{napoe\ (t2/t1)}$	-0.079	0.130	0.55	0.052	0.171	0.76
$PRS\_AD_{napoe\ (t3/t1)}$	0.005	0.130	0.97	−0.105	0.175	0.55
Age_{w1}(centered)	**0.083**	**0.016**	**<0.001**	**0.088**	**0.020**	**<0.001**
$PRS\_AD_{napoe\ (t2/t1)}$ x Age_{w1}(centered)	-0.005	0.022	0.80	0.023	0.028	0.41
$PRS_AD_{napoe\ (t3/t1)}$x Age_{w1}(centered)	0.013	0.022	0.55	0.026	0.029	0.36
$Age_{w1}(centered)^2$	0.002	0.001	0.20	0.001	0.002	0.50
$PRS\_AD_{napoe\ (t2/t1)}$ x $Age_{w1}(centered)^2$	0.003	0.002	0.12	0.002	0.002	0.41
$PRS_AD_{napoe\ (t3/t1)}$x $Age_{w1}(centered)^2$	0.002	0.002	0.35	0.004	0.003	0.17
Age_{w2}(centered)	**-0.168**	**0.014**	**<0.001**	**−0.174**	**0.016**	**<0.001**
$PRS\_AD_{napoe\ (t2/t1)}$ x Age_{w2}(centered)	-0.003	0.020	0.87	−0.013	0.023	0.55
$PRS\_AD_{napoe\ (t3/t1)}$ x Age_{w2}(centered)	-0.011	0.019	0.59	−0.001	0.023	0.98
$Age_{w2}(centered)^2$	**-0.005**	**0.001**	**<0.001**	**−0.005**	**0.001**	**<0.001**
$PRS\_AD_{napoe\ (t2/t1)}$ x $Age_{w2}(centered)^2$	-0.002	0.001	0.09	−0.003	0.002	0.09
$PRS\_AD_{napoe\ (t3/t1)}$ x $Age_{w2}(centered)^2$	-0.001	0.001	0.45	−0.001	0.002	0.55
$Sex_{Female/Male}$	**-0.397**	**0.087**	**<0.001**	**−0.243**	**0.113**	**0.03**
Educational year	**0.403**	**0.009**	**<0.001**	**0.380**	**0.012**	**<0.001**
$Smoking_{Current/Non+Former}$	-0.112	0.124	0.37	−0.321	0.165	0.05
PC_1	**-26.070**	**2.574**	**<0.001**	**−26.383**	**3.549**	**<0.001**
PC_2	**7.231**	**2.679**	**0.01**	**8.011**	**3.794**	**0.04**
PC_3	0.806	2.574	0.75	0.860	3.442	0.80
PC_4	1.723	2.633	0.51	2.172	4.067	0.59
Random effects						
Intercept	**4.026**	**0.150**	**<0.001**	**3.771**	**0.119**	**<0.001**
Residual	**3.947**	**0.098**	**<0.001**	**3.692**	**0.103**	**<0.001**

© 2026 Chung YCE et al. *JAMA Network Open*.

eTable 3. Mixed Models of PRS_ADnapoe as Continuous Variable Among Community-Dwellers Aged 55 Years or Older and Recruited From 2009 to 2013 in Taiwan (N = 4392)

	Overall (N = 4392)			Among participants with ε3 homozygotes (n=3082)		
Model parameters	Estimate	SE	P	Estimate	SE	P
Fixed effects						
Intercept	**41.900**	**13.410**	**0.002**	**71.680**	**22.490**	**0.001**
PRS_AD_{napoe}	-0.788	0.593	0.18	**-2.099**	**0.995**	**0.04**
Age_{w1}(centered)	-1.971	2.220	0.38	-3.278	3.691	0.38
PRS_AD_{napoe} x Age_{w1}(centered)	0.091	0.098	0.35	0.150	0.163	0.36
$Age_{w1}(centered)^2$	-0.308	0.174	0.08	-0.572	0.316	0.07
PRS_AD_{napoe} x $Age_{w1}(centered)^2$	0.014	0.008	0.07	0.025	0.014	0.07
Age_{w2}(centered)	1.840	1.966	0.35	0.825	3.006	0.78
PRS_AD_{napoe} x Age_{w2}(centered)	-0.089	0.087	0.31	-0.044	0.133	0.74
$Age_{w2}(centered)^2$	0.137	0.130	0.29	-0.020	0.203	0.92
PRS_AD_{napoe} x $Age_{w2}(centered)^2$	-0.006	0.006	0.27	0.001	0.009	0.95
$Sex_{Female/Male}$	**-0.394**	**0.087**	**<0.001**	**-0.238**	**0.113**	**0.04**
Educational year	**0.402**	**0.009**	**<0.001**	**0.380**	**0.012**	**<0.001**
$Smoking_{Current/Non+Former}$	-0.116	0.124	0.35	**-0.325**	**0.165**	**0.05**
PC_1	**-25.980**	**2.571**	**<0.001**	**-26.320**	**3.543**	**<0.001**
PC_2	**7.137**	**2.676**	**0.01**	**8.219**	**3.787**	**0.03**
PC_3	0.802	2.567	0.76	0.785	3.434	0.82
PC_4	1.634	2.630	0.53	1.791	4.059	0.66
Random effects						
Intercept	**4.023**	**0.105**	**<0.001**	**3.754**	**0.119**	**<0.001**
Residual	**3.945**	**0.124**	**<0.001**	**3.694**	**0.103**	**<0.001**

© 2026 Chung YCE et al. *JAMA Network Open*.

eTable 4. Examination of the Additive Effect Between *APOE* ε4 Carriage and PRS_ADnapoe via Likelihood Ratio Test Between 2 Models Among Community-Dwellers Aged 55 Years or Older and Recruited From 2009 to 2013 in Taiwan (N = 4392)

	Model1: *APOE ε4* carriage + PRS_AD_{napoe}			Model 2: *APOE ε4* carriage + PRS_AD_{napoe} + PRS_AD_{napoe} x *APOE ε4* carriage			The Likelihood Ratio Test between Model 1 &2
Model parameters	Estimate	SE	P	Estimate	SE	P	P
Fixed effects							0.49
Intercept	**24.040**	**0.188**	**<0.001**	**24.030**	**0.187**	**<0.001**	
Carrier	0.272	0.163	0.10	0.217	0.214	0.31	
PRS_AD_{napoe}	-0.117	0.061	0.06	-0.127	0.067	0.06	
Carrier x PRS_AD_{napoe}	-	-	-	0.059	0.161	0.71	
Age_{w1}(centered)	**0.078**	**0.010**	**<0.001**	**0.078**	**0.010**	**<0.001**	
Carrier x Age_{w1}(centered)	0.041	0.027	0.14	0.030	0.037	0.42	
PRS_AD_{napoe} x Age_{w1}(centered)	0.000	0.010	>0.99	-0.002	0.011	0.87	
Carrier x PRS_AD_{napoe} x Age_{w1}(centered)	-	-	-	0.013	0.027	0.64	
$Age_{w1}(centered)^2$	**0.003**	**0.001**	**<0.001**	**0.003**	**0.001**	**0.001**	
Carrier x $Age_{w1}(centered)^2$	0.001	0.002	0.79	-0.001	0.003	0.83	
PRS_AD_{napoe} x $Age_{w1}(centered)^2$	0.001	0.001	0.23	0.001	0.001	0.42	
Carrier x PRS_AD_{napoe} x $Age_{w1}(centered)^2$	-	-	-	0.001	0.002	0.52	
Age_{w2}(centered)	**-0.169**	**0.009**	**<0.001**	**-0.168**	**0.009**	**<0.001**	
Carrier x Age_{w2}(centered)	-0.021	0.024	0.40	0.008	0.033	0.81	
PRS_AD_{napoe} x Age_{w2}(centered)	-0.004	0.009	0.65	0.002	0.010	0.86	
Carrier x PRS_AD_{napoe} x Age_{w2}(centered)	-	-	-	-0.032	0.024	0.17	
$Age_{2}(centered)^2$	**-0.005**	**0.001**	**<0.001**	**-0.005**	**0.001**	**<0.001**	
Carrier x $Age_{w2}(centered)^2$	**-0.005**	**0.002**	**0.001**	**-0.005**	**0.002**	**0.02**	
PRS_AD_{napoe} x $Age_{w2}(centered)^2$	0.001	0.001	0.39	0.001	0.001	0.40	
Carrier x PRS_AD_{napoe} x $Age_{w2}(centered)^2$	-	-	-	0.000	0.002	0.83	
$Sex_{Female/Male}$	**-0.393**	**0.087**	**<0.001**	**-0.391**	**0.087**	**<0.001**	
Educational year	**0.402**	**0.009**	**<0.001**	**0.402**	**0.009**	**<0.001**	
$Smoking_{Current/Non+Former}$	-0.118	0.124	0.34	-0.119	0.124	0.34	
PC_1	**-25.910**	**2.570**	**<0.001**	**-26.050**	**2.565**	**<0.001**	
PC_2	**7.290**	**2.675**	**0.01**	**7.284**	**2.669**	**0.01**	
PC_3	0.734	2.570	0.78	0.685	2.565	0.79	
PC_4	1.418	2.628	0.59	1.435	2.623	0.58	
Random effects							
Intercept	4.013	**0.105**	**<0.001**	**3.990**	**0.105**	**<0.001**	
Residual	3.936	**0.124**	**<0.001**	**3.927**	**0.123**	**<0.001**	

© 2026 Chung YCE et al. *JAMA Network Open*.

eTable 5. Distribution of MMSE Scores Across the Total Sample, Participants With 1-Wave MMSE Only, and Participants With Complete 2-Wave MMSE

Variables	Sample	ε2ε2	ε2ε3	ε2ε4	ε3ε3	ε3ε4	ε4ε4	P-value
**Total sample, n**	**4392**	**26**	**528**	**55**	**3082**	**668**	**33**	
MMSE at Wave1, mean (SD)	26.5 (3.4)	27.3 (3.3)	26.3 (3.5)	27.0 (3.0)	26.6 (3.4)	26.5 (3.4)	27.0 (3.0)	0.22
**Baseline MMSE only, n**	**1133**	**8**	**137**	**14**	**809**	**154**	**11**	
MMSE at Wave1, mean (SD)	25.0 (3.9)	24.9 (4.4)	24.4 (4.1)	26.1 (3.5)	25.1 (3.9)	24.9 (3.9)	27.0 (2.9)	0.19
**Complete two-wave MMSE, n**	**3259**	**18**	**391**	**41**	**2273**	**514**	**22**	
Cross-sectional								
MMSE at Wave1, mean (SD)	27.1 (3.0)	28.4 (2.0)	26.9 (3.0)	27.3 (2.7)	27.1 (3.0)	27.0 (3.1)	27.0 (3.1)	0.35
MMSE at Wave2, mean (SD)	25.7 (4.1)	27.2 (2.2)	25.7 (4.1)	25.7 (5.0)	25.8 (4.0)	25.5 (4.3)	24.4 (6.4)	0.24
Longitudinal								
Change in MMSE, mean (SD)	-1.3 (2.9)	-1.3 (2.8)	-1.2 (3.0)	-1.6 (3.0)	-1.3 (2.8)	-1.5 (3.0)	-2.6 (4.6)	0.20
Annual change in MMSE, mean (SD)	-0.2 (0.5)	-0.2 (0.5)	-0.2 (0.5)	-0.3 (0.5)	-0.2 (0.4)	-0.2 (0.5)	-0.4 (0.8)	0.14

© 2026 Chung YCE et al. *JAMA Network Open*.

eTable 6. Mixed Models of Different *APOE* Genotypes Among Community-Dwellers Aged 55 Years or Older and Recruited From 2009 to 2013 in Taiwan (N = 4392).

Model parameters	Estimate	SE	P
Fixed effects			
**Intercept**	**24.056**	**0.189**	**<0.001**
ε2ε3	-0.049	0.167	0.77
ε2ε4	-0.270	0.496	0.59
ε3ε4	0.152	0.151	0.31
ε2ε2	0.799	0.625	0.20
ε4ε4	0.361	0.722	0.62
**Age_{w1}(centered)**	**0.079**	**0.011**	**<0.001**
ε2ε3 x Age_{w1}(centered)	-0.008	0.027	0.77
ε2ε4 x Age_{w1}(centered)	0.011	0.081	0.90
ε3ε4 x Age_{w1}(centered)	0.039	0.025	0.12
ε2ε2 x Age_{w1}(centered)	-0.021	0.115	0.86
ε4ε4 x Age_{w1}(centered)	0.136	0.109	0.21
**Age_{w1}(centered)^2**	**0.003**	**0.001**	**0.001**
ε2ε3 x Age_{w1}(centered)^2	-0.002	0.002	0.35
ε2ε4 x Age_{w1}(centered)^2	0.011	0.007	0.10
ε3ε4 x Age_{w1}(centered)^2	0.000	0.002	0.90
ε2ε2 x Age_{w1}(centered)^2	-0.001	0.007	0.93
ε4ε4 x Age_{w1}(centered)^2	0.014	0.012	0.26
**Age_{w2}(centered)**	**-0.169**	**0.009**	**<0.001**
ε2ε3 x Age_{w2}(centered)	0.006	0.024	0.82
ε2ε4 x Age_{w2}(centered)	0.018	0.074	0.81
ε3ε4 x Age_{w2}(centered)	-0.021	0.022	0.33
ε2ε2 x Age_{w2}(centered)	-0.003	0.100	0.97
**ε4ε4 x Age_{w2}(centered)**	**-0.213**	**0.095**	**0.03**
**Age_{w2}(centered)^2**	**-0.006**	**0.001**	**<0.001**
ε2ε3 x Age_{w2}(centered)^2	0.002	0.002	0.31
ε2ε4 x Age_{w2}(centered)^2	-0.006	0.004	0.15
**ε3ε4 x Age_{w2}(centered)^2**	**-0.004**	**0.001**	**0.006**
ε2ε2 x Age_{w2}(centered)^2	0.001	0.008	0.94
**ε4ε4 x Age_{w2}(centered)^2**	**-0.017**	**0.008**	**0.03**
**$Sex_{Female/Male}$**	**-0.392**	**0.087**	**<0.001**
**Educational year**	**0.402**	**0.009**	**<0.001**
$Smoking_{Current/Non+Former}$	-0.115	0.124	0.36
**PC_1**	**-25.926**	**2.572**	**<0.001**
**PC_2**	**7.008**	**2.677**	**0.01**
PC_3	0.563	2.570	0.83

© 2026 Chung YCE et al. *JAMA Network Open*.

Model parameters	Estimate	SE	P
PC_4	1.769	2.630	0.50
Random effects			
**Intercept**	**4.022**	**0.150**	**<0.001**
**Residual**	**3.934**	**0.098**	**<0.001**

eTable 7. Sensitivity Analysis for Association Between *APOE* ε4 Carriers and Noncarriers and MMSE Score Change in Different Scenarios

	Scenario 1: Exclude participants with one-wave MMSE only (n=3259)			Scenario 2: Exclude participants with any item missing in either wave of MMSE (n=2468)			Scenario 3: Change MMSE cut point: exclude participants with MMSE < 21 (n=4028)			Scenario 4: Change MMSE cut point: exclude participants with MMSE < 24 (n=3590)			Scenario 5: Adjust the model with inverse probability of censoring weighting (N=4392)		
Model Parameters	Estimate	SE	P	Estimate	SE	P	Estimate	SE	P	Estimate	SE	P	Estimate	SE	P
Fixed effects															
Intercept	**24.390**	**0.211**	**<0.001**	**28.140**	**0.148**	**<0.001**	**25.100**	**0.184**	**<0.001**	**26.070**	**0.160**	**<0.001**	**24.090**	**0.193**	**<0.001**
Carrier	0.101	0.153	0.51	0.035	0.127	0.79	-0.039	0.142	0.78	0.108	0.127	0.40	0.130	0.145	0.37
Age_{w1}(centered)	**0.096**	**0.011**	**<0.001**	**0.071**	**0.010**	**<0.001**	**0.118**	**0.011**	**<0.001**	**0.131**	**0.010**	**<0.001**	**0.084**	**0.01**	**<0.001**
Carrier x Age_{w1}(centered)	0.029	0.026	0.26	0.015	0.024	0.52	0.022	0.025	0.37	0.029	0.024	0.23	0.041	0.024	0.09
$Age_{w1}(centered)^2$	**0.003**	**0.001**	**0.007**	0.002	0.001	0.07	**0.003**	**0.001**	**0.001**	**0.003**	**0.001**	**<0.001**	**0.003**	**0.001**	**<0.001**
Carrier x $Age_{w1}(centered)^2$	0.001	0.002	0.67	-0.001	0.002	0.66	0.002	0.002	0.33	0.001	0.002	0.49	0.002	0.002	0.36
Age_{w2}(centered)	**-0.161**	**0.009**	**<0.001**	**-0.132**	**0.009**	**<0.001**	**-0.177**	**0.009**	**<0.001**	**-0.175**	**0.009**	**<0.001**	**-0.173**	**0.009**	**<0.001**
Carrier x Age_{w2}(centered)	-0.015	0.022	0.50	-0.008	0.021	0.71	-0.022	0.021	0.30	-0.013	0.021	0.55	-0.027	0.021	0.21
$Age_{w2}(centered)^2$	**-0.006**	**0.001**	**<0.001**	**-0.003**	**0.001**	**<0.001**	**-0.006**	**0.001**	**<0.001**	**-0.006**	**0.001**	**<0.001**	**-0.006**	**0.001**	**<0.001**
Carrier x $Age_{w2}(centered)^2$	**-0.004**	**0.001**	**0.003**	-0.001	0.001	0.56	**-0.003**	**0.001**	**0.05**	-0.002	0.001	0.08	**-0.005**	**0.001**	**<0.001**
Sex $_{Female/Male}$	**-0.291**	**0.095**	**0.002**	**-0.213**	**0.073**	**0.004**	-0.142	0.083	0.09	-0.030	0.071	0.67	**-0.430**	**0.09**	**<0.001**
Educational year	**0.384**	**0.010**	**<0.001**	**0.045**	**0.005**	**<0.001**	**0.303**	**0.009**	**<0.001**	**0.217**	**0.008**	**<0.001**	**0.406**	**0.009**	**<0.001**
Smoking $_{Current/Non+Former}$	-0.237	0.142	0.10	**-0.307**	**0.111**	**0.01**	-0.182	0.124	0.14	-0.197	0.105	0.06	-0.150	0.127	0.24
PC_1	**-27.250**	**2.842**	**<0.001**	**-20.430**	**3.095**	**<0.001**	**-20.400**	**2.866**	**<0.001**	**-14.410**	**2.833**	**<0.001**	**-25.950**	**2.651**	**<0.001**
PC_2	**8.284**	**3.102**	**0.01**	**5.618**	**2.347**	**0.02**	**8.136**	**2.705**	**0.003**	**8.229**	**2.286**	**<0.001**	**7.134**	**2.745**	**0.009**
PC_3	1.324	2.883	0.65	**4.786**	**2.350**	**0.04**	2.736	2.548	0.28	3.351	2.204	0.13	0.918	2.646	0.73
PC_4	2.103	3.448	0.54	-1.155	3.125	0.71	0.654	2.991	0.83	-0.169	2.536	0.95	1.694	2.696	0.53

© 2026 Chung YCE et al. *JAMA Network Open*.

Model Parameters	Scenario 1: Exclude participants with one-wave MMSE only (n=3259)			Scenario 2: Exclude participants with any item missing in either wave of MMSE (n=2468)			Scenario 3: Change MMSE cut point: exclude participants with MMSE < 21 (n=4028)			Scenario 4: Change MMSE cut point: exclude participants with MMSE < 24 (n=3590)			Scenario 5: Adjust the model with inverse probability of censoring weighting (N=4392)		
	Estimate	SE	P	Estimate	SE	P	Estimate	SE	P	Estimate	SE	P	Estimate	SE	P
Random effects															
Intercept	**3.773**	**0.150**	**<0.001**	**1.459**	**0.062**	**<0.001**	**2.334**	**0.078**	**<0.001**	**1.218**	**0.071**	**<0.001**	**4.398**	**0.106**	**<0.001**
Residual	**3.875**	**0.096**	**<0.001**	**2.657**	**0.070**	**<0.001**	**3.717**	**0.089**	**<0.001**	**3.263**	**0.083**	**<0.001**	**3.957**	**0.123**	**<0.001**

© 2026 Chung YCE et al. *JAMA Network Open*.

eTable 8. Sensitivity Analysis for Association Between *APOE* ε4 Homozygous, Heterozygous, and Noncarrier Status and MMSE Score Change in Different Scenarios

	Scenario 1: Exclude participants with one-wave MMSE only (n=3259)			Scenario 2: Exclude participants with any item missing in MMSE (n=2627)			Scenario 3: Change MMSE cut point: exclude participants with MMSE < 21 (n=4028)			Scenario 4: Change MMSE cut point: exclude participants with MMSE < 24 (n=3590)			Scenario 5: Adjust the model with inverse probability of censoring weighting (N=4392)		
Model Parameters	Estimate	SE	P	Estimate	SE	P	Estimate	SE	P	Estimate	SE	P	Estimate	SE	P
Fixed effects															
Intercept	**24.370**	**0.211**	**<0.001**	**28.130**	**0.148**	**<0.001**	**25.090**	**0.186**	**<0.001**	**26.050**	**0.162**	**<0.001**	**24.080**	**0.193**	**<0.001**
Hetero	0.117	0.155	0.45	0.031	0.129	0.81	0.052	0.176	0.77	0.082	0.158	0.61	0.135	0.147	0.36
Homo	0.149	0.764	0.85	0.646	0.691	0.35	-0.003	0.142	0.98	0.142	0.127	0.27	0.256	0.737	0.73
Age_{w1}(centered)	**0.096**	**0.011**	**<0.001**	**0.072**	**0.010**	**<0.001**	**0.121**	**0.012**	**<0.001**	**0.130**	**0.011**	**<0.001**	**0.084**	**0.01**	**<0.001**
Hetero x Age_{w1}(centered)	0.029	0.026	0.27	0.015	0.024	0.54	-0.019	0.029	0.51	0.004	0.028	0.89	0.038	0.025	0.13
Homo x Age_{w1}(centered)	0.016	0.129	0.90	0.048	0.117	0.68	0.018	0.025	0.48	0.029	0.024	0.22	0.156	0.11	0.16
Age_{w1}(centered)^2	**0.003**	**0.001**	**0.01**	0.002	0.001	0.07	**0.004**	**0.001**	**<0.001**	**0.004**	**0.001**	**<0.001**	**0.003**	**0.001**	**<0.001**
Hetero x Age_{w1}(centered)^2	0.000	0.002	0.84	-0.001	0.002	0.66	-0.003	0.003	0.19	-0.004	0.002	0.14	0.001	0.002	0.47
Homo x Age_{w1}(centered)^2	0.011	0.016	0.49	-0.008	0.013	0.54	0.002	0.002	0.47	0.001	0.002	0.60	0.014	0.012	0.24
Age_{w2}(centered)	**-0.161**	**0.009**	**<0.001**	**-0.132**	**0.009**	**<0.001**	**-0.176**	**0.010**	**<0.001**	**-0.174**	**0.010**	**<0.001**	**-0.173**	**0.009**	**<0.001**
Hetero x Age_{w2}(centered)	-0.009	0.023	0.69	-0.007	0.021	0.76	-0.002	0.025	0.95	-0.008	0.025	0.74	-0.019	0.022	0.38
Homo x Age_{w2}(centered)	-0.166	0.102	0.10	-0.053	0.099	0.59	-0.022	0.021	0.30	-0.014	0.021	0.50	**-0.229**	**0.094**	**0.02**
Age_{w2}(centered)^2	**-0.006**	**0.001**	**<0.001**	**-0.003**	**0.001**	**<0.001**	**-0.006**	**0.001**	**<0.001**	**-0.006**	**0.001**	**<0.001**	**-0.006**	**0.001**	**<0.001**
Hetero x Age_{w2}(centered)^2	**-0.004**	**0.001**	**0.01**	-0.001	0.001	0.64	0.001	0.002	0.45	0.002	0.002	0.21	**-0.005**	**0.001**	**<0.001**
Homo x Age_{w2}(centered)^2	-0.014	0.008	0.10	-0.010	0.008	0.18	-0.003	0.001	0.07	-0.002	0.001	0.11	**-0.015**	**0.008**	**0.05**
$Sex_{Female/Male}$	**-0.283**	**0.095**	**0.003**	**-0.208**	**0.073**	**0.004**	-0.140	0.083	0.09	-0.028	0.071	0.69	**-0.426**	**0.09**	**<0.001**
Educational year	**0.385**	**0.010**	**<0.001**	**0.045**	**0.005**	**<0.001**	**0.302**	**0.009**	**<0.001**	**0.217**	**0.008**	**<0.001**	**0.407**	**0.009**	**<0.001**

© 2026 Chung YCE et al. *JAMA Network Open*.

Model Parameters	Scenario 1: Exclude participants with one-wave MMSE only (n=3259)			Scenario 2: Exclude participants with any item missing in MMSE (n=2627)			Scenario 3: Change MMSE cut point: exclude participants with MMSE < 21 (n=4028)			Scenario 4: Change MMSE cut point: exclude participants with MMSE < 24 (n=3590)			Scenario 5: Adjust the model with inverse probability of censoring weighting (N=4392)		
	Estimate	SE	P	Estimate	SE	P	Estimate	SE	P	Estimate	SE	P	Estimate	SE	P
Smoking $_{Current/Non+Former}$	-0.232	0.142	0.10	**-0.305**	**0.111**	**0.01**	-0.178	0.124	0.15	-0.194	0.106	0.07	-0.146	0.127	0.25
PC_1	**-27.250**	**2.840**	**<0.001**	**-20.400**	**3.094**	**<0.001**	**-20.390**	**2.868**	**<0.001**	**-14.380**	**2.835**	**<0.001**	**-25.950**	**2.652**	**<0.001**
PC_2	**8.399**	**3.102**	**0.01**	**5.719**	**2.348**	**0.02**	**8.114**	**2.707**	**0.003**	**8.211**	**2.288**	**<0.001**	**7.238**	**2.747**	**0.01**
PC_3	1.256	2.884	0.66	**4.651**	**2.351**	**0.05**	2.756	2.550	0.28	3.359	2.206	0.13	0.823	2.649	0.76
PC_4	2.138	3.446	0.54	-1.153	3.125	0.71	0.601	2.996	0.84	-0.093	2.541	0.97	1.755	2.697	0.52
Random effects															
Intercept	**3.769**	**0.150**	**<0.001**	**1.459**	**0.062**	**<0.001**	**2.337**	**0.078**	**<0.001**	**1.220**	**0.071**	**<0.001**	**4.403**	**0.106**	**<0.001**
Residual	**3.871**	**0.096**	**<0.001**	**2.656**	**0.070**	**<0.001**	**3.718**	**0.089**	**<0.001**	**3.262**	**0.084**	**<0.001**	**3.956**	**0.123**	**<0.001**

© 2026 Chung YCE et al. *JAMA Network Open*.
