# Supplementary material for: APOE ε4 and Accelerated Cognitive Decline Among Cognitively Healthy Middle-Aged and Older Adults
Source: JAMA Netw Open. 2026 Mar 6;9(3):e260853. doi: 10.1001/jamanetworkopen.2026.0853 (PMC12966930; doi:10.1001/jamanetworkopen.2026.0853)
Supplement: Supplement 2. — Data Sharing Statement [file jamanetwopen-e260853-s002.pdf]

Data Sharing Statement

Chung. APOE ε4 and Accelerated Cognitive Decline Among Cognitively Healthy Middle-Aged and Older Adults. *JAMA Netw Open*. Published March 06, 2026.
doi:10.1001/jamanetworkopen.2026.0853

Data
Data available: Yes
Data types: Deidentified participant data
How to access data: The datasets analyzed during the current study are available from the corresponding author on reasonable request
When available: With publication

Supporting Documents
Document types: Statistical/analytic code
How to access documents: The corresponding author
When available: With publication

Additional Information
Who can access the data: researchers whose proposed use of the data has been approved
Types of analyses: for a specified purpose
Mechanisms of data availability: with investigator support
Any additional restrictions: None.
